# Modeling the Impact of Phonon Scattering with Strain Effects on the Electrical Properties of MoS_2_ Field-Effect Transistors

**DOI:** 10.3390/mi14061235

**Published:** 2023-06-12

**Authors:** Huei Chaeng Chin, Afiq Hamzah, Nurul Ezaila Alias, Michael Loong Peng Tan

**Affiliations:** Faculty of Electrical Engineering, Universiti Teknologi Malaysia, Skudai 81310, Johor, Malaysia; hcchin3@graduate.utm.my (H.C.C.); mafiq@utm.my (A.H.); ezaila@utm.my (N.E.A.)

**Keywords:** MoS_2_ FET, phonon, acoustic, optical, mean free path, strain, I–V

## Abstract

Molybdenum disulfide (MoS_2_) has distinctive electronic and mechanical properties which make it a highly prospective material for use as a channel in upcoming nanoelectronic devices. An analytical modeling framework was used to investigate the I–V characteristics of field-effect transistors based on MoS_2_. The study begins by developing a ballistic current equation using a circuit model with two contacts. The transmission probability, which considers both the acoustic and optical mean free path, is then derived. Next, the effect of phonon scattering on the device was examined by including transmission probabilities into the ballistic current equation. According to the findings, the presence of phonon scattering caused a decrease of 43.7% in the ballistic current of the device at room temperature when *L* = 10 nm. The influence of phonon scattering became more prominent as the temperature increased. In addition, this study also considers the impact of strain on the device. It is reported that applying compressive strain could increase the phonon scattering current by 13.3% at *L* = 10 nm at room temperature, as evaluated in terms of the electrons’ effective masses. However, the phonon scattering current decreased by 13.3% under the same condition due to the existence of tensile strain. Moreover, incorporating a high-k dielectric to mitigate the impact of scattering resulted in an even greater improvement in device performance. Specifically, at *L* = 6 nm, the ballistic current was surpassed by 58.4%. Furthermore, the study achieved SS = 68.2 mV/dec using Al_2_O_3_ and an on–off ratio of 7.75 × 10^4^ using HfO_2_. Finally, the analytical results were validated with previous works, showing comparable agreement with the existing literature.

## 1. Introduction

Silicon-based metal oxide semiconductor field-effect transistors (MOSFETs) have been widely used in the last few decades. However, as the channel length has been shrinking, short channel effects [1,2] have emerged and decreased the device’s precision. Two-dimensional (2D) materials have emerged as promising candidates to tackle these issues. In 2004, the discovery of graphene [3] drew the attention of researchers to study the potential of 2D materials, such as boron phosphide [4], black phosphorus [5] and silicene [6], for use as channels in devices. Molybdenum disulfide (MoS_2_) is also a member of the 2D material family and is being considered as a future channel material to solve scaling issues. The realization of the first semiconductor device based on an MoS_2_-based monolayer took placed in 2005 [7]. MoS_2_ is already widely studied in applications, including image sensors [8], solar cells [9], photosensitizers [10], optoelectronic memory devices [11] and valleytronic devices [12].

Several studies have been conducted to explore the potential of using MoS_2_ as a replacement for SiO_2_ as a channel material. Ahmed et al. [13] introduced a MoS_2_ FET with both short and long channel lengths, demonstrating its comparability to traditional Si transistors. Shunli et al. [14] designed a highly accurate SPICE model specifically tailored for MoS_2_ FET. This model accounts for the non-idealities of the channel material and considers the impact of Schottky contact. In addition, Nandan et al. [15] proposed a double gate MoS_2_ FET which considered the effect of channel thickness. Additionally, Khare et al. [16] reported that the threshold voltage of MoS_2_ FET is influenced by the layer’s thickness. Subsequently, Singh et al. [17] established a model for short channel TMD FET that included the effect of source-to-drain tunneling. Moreover, Zeng et al. [18] proposed a compact model for MoS_2_ FET that considers the effect of trap charges. Apart from this, Ehsan et al. [19] created a 5 nm ballistic MoS_2_ FET SPICE model based on Natori’s theory of ballistic MOSFET. Furthermore, Silvestri et al. [20] developed a hierarchical modeling methodology specifically designed for a short channel ballistic model of TMD FET. Then, Haixia et al. [21] demonstrated that MoS_2_ FET can achieve SS = 70 mv/dec. However, these studies did not consider the effects of phonon scattering and strain in their models.

Various theoretical and experimental studies have investigated the effects of phonon scattering and strain on MoS_2_ FET. Tiwari et al. [22] found that the impact of optical phonons decreases as temperature increases, with a current variation of about 6.7% and between 300 K and 400 K. Pilotto et al. [23] demonstrated that optical phonons have a significant effect on the current. In addition, Guo et al. [24] showed that scattering is significant at higher temperatures. Meanwhile, strain has been shown to enhance the performance of MoS_2_ FET. Khair et al. [25] suggested that optical phonons became dominant at higher temperatures but can be suppressed by applying strain, which can increase the on-current by about 15.56%. Next, Chai et al. [26] claimed that strain can raise the on current by 46%, indicating a positive effect on the device. Peto et al. [27] discovered that effective mass decreases with increasing strain, enhancing the conductivity of MoS_2_. Subsequently, Chen et al. [28] reported that tensile strain can increase carrier mobility by two orders of magnitude and improve the performance of MoS_2_. Finally, Kaushal et al. [29] reported similar results to [27] by showing the effect of tensile strain on the effective mass of different MoS_2_ structures.

In this study, we present an analytical I–V model for MoS_2_ FET, which extends the FETToy framework [30]. Specifically, we include the impact of phonon scattering and strain in our model based on the findings of prior research [19]. Section 2 discusses the formulation of the model, and the results are presented in Section 3. Finally, Section 4 has our conclusions.

## 2. Device Modeling

**Figure 1:**
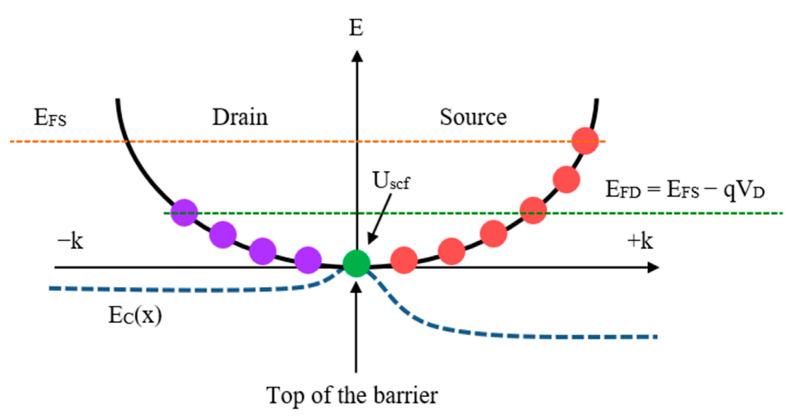
A visual representation illustrating the process of filling electrons in the k states under non-equilibrium conditions.

The electrons located at the top of the barriers are filled either from the source or drain regions. Electrons from the source region possess positive velocities, while electrons from the drain region possesses negative velocities. Consequently, the electrons originating from the source occupy the positive velocity states (+k), while those from the drain occupy the negative velocity states (−k). The population of electrons in the +k state is determined by the source Fermi level, $E_{FS}$, whereas the population in the −k state is determined by the drain Fermi level, $E_{FD}$. Under equilibrium conditions, an equal number of electrons occupy both states. The equilibrium electron density is achieved when the biases are set to zero [31]:

(1) $$N_{0}=\int_{-\infty }^{+\infty }D\left(E\right)f\left(E-E_{F}\right)dE,$$

where $D\left(E\right)$ is the density of states of the channel and $f\left(E-E_{F}\right)$ is the equilibrium Fermi function. Both the source and drain regions contribute equally to the available states, and they populate their respective halves based on the Fermi level. However, when a drain bias is applied, the states at the top of the barrier are now populated by two distinct Fermi levels. Consequently, the electron density at the +k states and −k states is affected, which is represented by [32]:

(2) $$N_{S}=\frac{1}{2}\int_{-\infty }^{+\infty }D\left(E-U_{scf}\right)f\left(E-E_{FS}\right)dE,$$

(3) $$N_{D}=\frac{1}{2}\int_{-\infty }^{+\infty }D\left(E-U_{scf}\right)f\left(E-E_{FD}\right)dE,$$

where $U_{scf}$ is the self-consistent potential, $E_{FS}=E_{F}$ and $E_{FD}=E_{F}-qV_{D}$. Due to the decrease in the barrier height at the drain side by $qV_{D}$, electrons begin to flow from the source to the drain, as shown in Figure 1. Simultaneously, some low-energy electrons are reflected back to the source and occupy the +k states again. In simpler terms, this implies that there is a flow of current.

**Figure 2:**
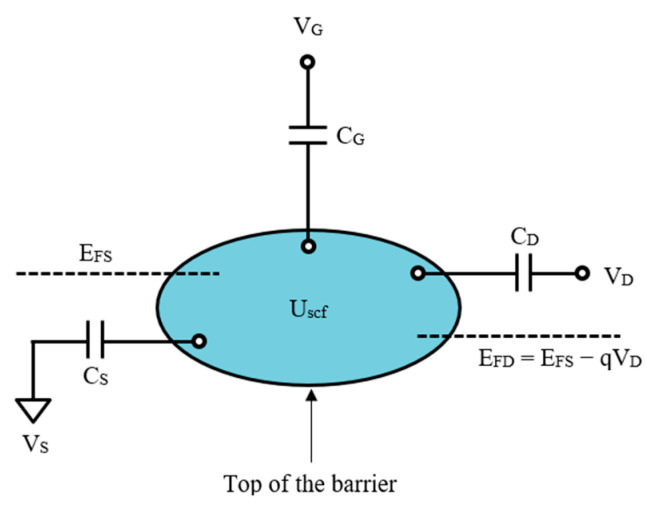
Schematic diagram of ballistic nanotransistor circuit model.

An analytical circuit model consisting of two contacts is depicted in Figure 2. The source, located on the left, is always connected to the ground and has a Fermi level of $E_{FS}$. The right contact, or drain, has a Fermi level of $E_{FD}=E_{FS}-qV_{D}$, where *q* is the electron charge and *V_D_* is the drain voltage. The two Fermi levels are in the same equilibrium condition but differ when a bias is applied. A gate, which is the third contact, is used to alter the energy states of the device through $U_{scf}$ [30,32].

(4) $$U_{scf}=-\frac{q}{C_{\Sigma }}\left[Q_{T}+q\Delta N\right],$$

where $C_{\Sigma }=C_{G}+C_{D}+C_{S}$ is the total capacitance, $Q_{T}=C_{G}V_{G}+C_{D}V_{D}+C_{S}V_{S}$ is the total charge at the terminals and ΔN is bias induced charge where $\Delta N=N_{S}+N_{D}-N_{0}$. Re-expressing Equations (2) and (3),

(5) $$N_{S}=\frac{1}{2}\int_{-\infty }^{+\infty }D\left(E\right)f_{S}\left(E\right)dE,$$

(6) $$N_{D}=\frac{1}{2}\int_{-\infty }^{+\infty }D\left(E\right)f_{D}\left(E\right)dE,$$

where $f_{S}\left(E\right)\equiv f\left(E+U_{scf}-E_{FS}\right)$ and $f_{D}\left(E\right)\equiv f\left(E+U_{scf}-E_{FD}\right)$. Meanwhile, $D\left(E\right)$ for MoS_2_ [33] is represented by the following equation:

(7) $$D\left(E\right)=\frac{g_{K}m_{K}^{*}k_{B}T}{\pi \hbar^{2}}+\frac{g_{Q}m_{Q}^{*}k_{B}T}{\pi \hbar^{2}}exp^{-\frac{\Delta E_{KQ}}{k_{B}T}},$$

where $g_{K}=2$ and $g_{Q}=6$ are the degeneracy of the *K* and *Q* conduction valleys, respectively. The terms $m_{K}^{*}=0.48m_{0}$ and $m_{Q}^{*}=0.57m_{0}$ are their respective density of state effective masses [34]. The term $\Delta E_{KQ}$ is the energy separation between *K* and *Q* conduction valleys, which is around 2k_B_T for MoS_2_ [35]. Further valleys are not taken into account since they are too far to contribute to electric conduction under normal bias conditions [36].

By solving N and $U_{scf}$, the drain current, $I_{D}$, is calculated using the Fermi–Dirac statistic, and it is denoted as [37]:

(8) $$I_{D}=\frac{q}{2}v_{avg}\int_{-\infty }^{+\infty }D\left(E\right)\left[f_{S}\left(E\right)-f_{D}\left(E\right)\right]dE,$$

where $\upsilon_{avg}=\sqrt{\frac{2k_{B}T}{\pi m^{*}}}$ is a thermal average velocity.

### 2.1. Phonon Mean Free Path

The transport of electrons is primarily determined by the channel length, L. In the case where L is significantly larger than the mean free path (MFP), $\lambda_{0}$, $\left(L\gg \lambda_{0}\right)$, electron transport follows a drift-diffusive pattern with notable scattering effects. Conversely, when L is approximately equal to $\lambda_{0}$, $\left(L\approx \lambda_{0}\right)$, electrons exhibit quasi-ballistic transport with minimal scattering effects. Last, if L is scaled down to be much smaller than $\lambda_{0}$, $\left(L\ll \lambda_{0}\right)$, electrons undergo fully ballistic transport without encountering scattering effects [38]. Physically, $\lambda_{0}$ represents the average distance that electrons travel before experiencing scattering events or collision with other electrons, which can alter their direction of motion [39]. In short, a ballistic transistor is realized when *L* is comparable with $\lambda_{0}$. However, 2D material-based FETs generally have higher electron phonon scattering rates than their 3D counterparts due to the higher density of phonons that can interact with the electrons [40]. This effect is also influenced by the strong interaction between the electrons in the conduction band and the holes in the valence band [41]. Two types of phonons are present, namely acoustic and optical [42]. Consider a unit cell with two atoms of masses, $M_{1}$ and $M_{2}$ that vibrate at their equilibrium positions: Acoustical vibration occurs when both atoms shift either to the left or right. Meanwhile, optical vibration occurs when the two atoms move away from each other in opposite directions [43].

### 2.2. Transmission Probability

**Figure 3:**
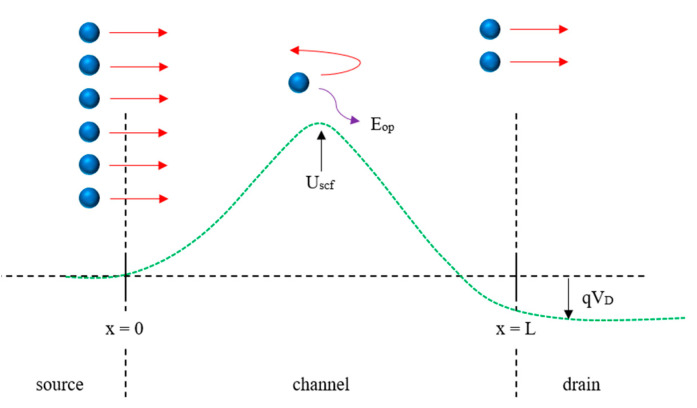
A simple diagram to illustrate the phonon scattering effects.

The phonon scattering effects have hindered the realization of the perfect device. Consider a device with *L* as depicted in Figure 3. At the starting point *x* = 0, only the electrons coming from the source can enter the channel. As they travel some distance (*x* < *L*), some of the electrons may lose energy, which is the energy of the optical phonon, *E_op_*, and is reflected back to the source. The existence of phonon scattering in a device is characterized by the transmission probability, $T_{r}$.

(9) $$T_{r}=\frac{\lambda_{0}}{\lambda_{0}+L}.$$

When $L\ll \lambda_{0}$, $T_{r}$ is approaching 1 and vice versa, the impact of phonon scattering is integrated into the device by adding $T_{r}$ into (5). $T_{S}$ ($T_{D}$) is the source side (drain side) transmission probability, which is computed as follows [44]:

(10) $$T_{S}\left(E\right)=\frac{L_{eff}\left(0\right)}{L_{eff}\left(0\right)+L},$$

(11) $$T_{D}\left(E\right)=\frac{L_{eff}\left(V_{DS}\right)}{L_{eff}\left(V_{DS}\right)+L}.$$

$L_{eff}$ is computed as follows:

(12) $$\frac{1}{L_{eff}}=\frac{1}{L_{ac}}\left(1-\frac{1}{1+exp^{\left(E_{F}-U_{scf}+qV_{D}\right)/k_{B}T}}\right)+\frac{1}{L_{op}}\left(1-\frac{1}{1+exp^{\left(E_{F}-U_{scf}-E_{op}+qV_{D}\right)/k_{B}T}}\right).$$

The values of *L_ac_* = 18.1 nm [45] and *E_op_* = 0.048 eV [22] for MoS_2_; *L_op_* is calculated as follows [46]:

(13) $$L_{op}=\frac{r_{b}\varepsilon_{\infty }}{\varepsilon_{0}-\varepsilon_{\infty }},$$

where *r_b_* = 0.053 nm is the effective Bohr radius, and *ε*_∞_ = 15.1 (*ε*_0_ = 15.3) is the optical (static) dielectric constant [47] for MoS_2_. Thus, *L_op_* = 4 nm.

Hence, (8) is redefined as follows:

(14) $$I_{D}=\frac{q}{2}v_{avg}\int_{-\infty }^{+\infty }D\left(E\right)\left[T_{S}\left(E\right)f_{S}\left(E\right)-T_{D}\left(E\right)f_{D}\left(E\right)\right]dE.$$

## 3. Results and Discussion

**Figure 4:**
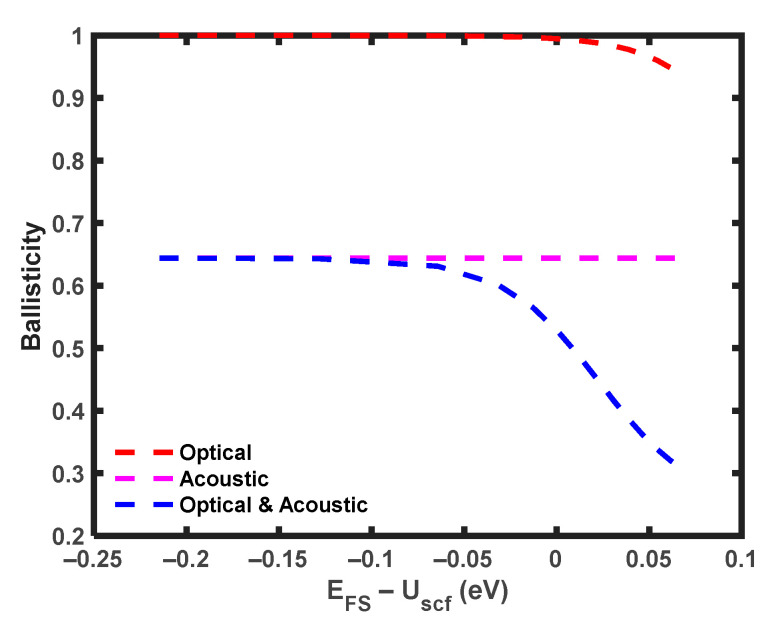
Ballisticity of MoS_2_ FET with *L* = 10 nm at *V_D_* = 0.5 V at T = 300 K.

The parameter used to determine the extent of phonon scattering effects in the device is referred to as ballisticity. It is calculated by taking the ratio of the phonon scattering current to the ballistic current. The ballisticity is computed by setting the *L_op_* value to infinity in Equation (12) for the scenario of acoustic phonon scattering only. Similarly, for the scenario of optical phonon scattering only, the ballisticity is determined by setting the *L_ac_* value to infinity in Equation (12). Figure 4 illustrates the impact of acoustic and optical phonon scattering on ballisticity. At low energy levels (*E_FS_* − *U_scf_* < 0), acoustic phonon scattering significantly affects the ballisticity, resulting in a value of only 0.644. Conversely, the ballisticity associated with optical phonon scattering approaches that of a ballistic device, reaching approximately one. This is attributed to the limited number of high-energy electrons that can transverse the high barrier and reach the drain without backscattering to the source, leading to a higher ballisticity. As the gate bias increases (*E_FS_* − *U_scf_* > 0), the influence of optical phonon scattering becomes more significant [48], and the ballisticity aligns with the trend of optical phonon scattering. At *V_G_* = 0.6 V, the impact of optical phonon scattering surpasses that of acoustic phonon scattering, resulting in a sudden drop in ballisticity. This phenomenon arises from the increased tendency of electrons to scatter before reaching the drain, attributed to the shorter MFP of optical phonons. Moreover, the lower potential barrier facilitates the electrons’ return to the source, ultimately resulting in reduced ballisticity. It is important to note that while optical phonons attempt to maintain ballisticity at low energy levels, the presence of acoustic phonons causes a sudden decline in ballisticity at all energy levels.

**Figure 5:**
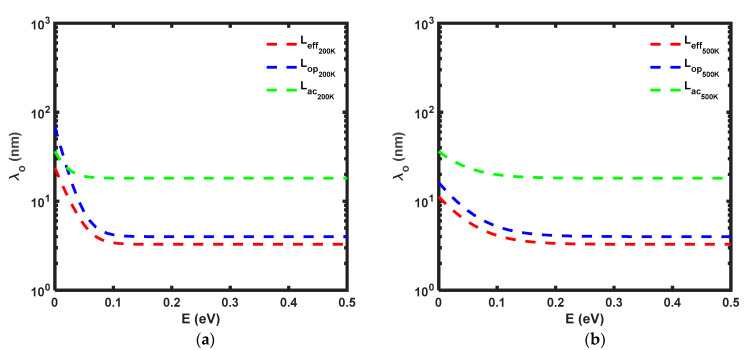
MFP of MoS_2_ at (**a**) T = 200 K (**b**) T = 500 K.

Theoretically, electrons can acquire energy through both thermal and electrical means. In Figure 5, MFP decreases as the energy increases, regardless of the temperature. At low energy and low temperatures (200 K), *L_eff_* is predominantly influenced by *L_ac_*, indicating that *L_op_* does not play a significant role in determining *L_eff_*. This indicates that at 200 K, electrons primarily interact with acoustic phonons since they have not yet acquired the minimum energy, *E_op_*, required for optical phonon scattering. Additionally, due to the high potential barrier, most of the electrons are reflected back to the source. Therefore, *L_ac_* dominates the determination of *L_eff_*. However, as the energy increases, *L_op_* begins to surpass the role of *L_ac_*. For E ≥ 0.03 V, *L_eff_* is determined by *L_op_*, indicating that the electrons have reached the *E_op_*_,_ and *L_op_* takes over control of *L_eff_*. Furthermore, at E ≥ 0.05 V, *L_op_* becomes one order lower than *L_ac_*. Eventually, *L_eff_* reaches a constant value of 3.28 nm at E ≥ 0.15 V. On the other hand, at 500 K, regardless of the energy levels, *L_op_* directly controls *L_eff_* as the electrons have thermally acquired sufficient energy to pass through the potential barrier. Finally, *L_eff_* becomes constant at E ≥ 0.3 V, remaining at 3.28 nm.

**Figure 6:**
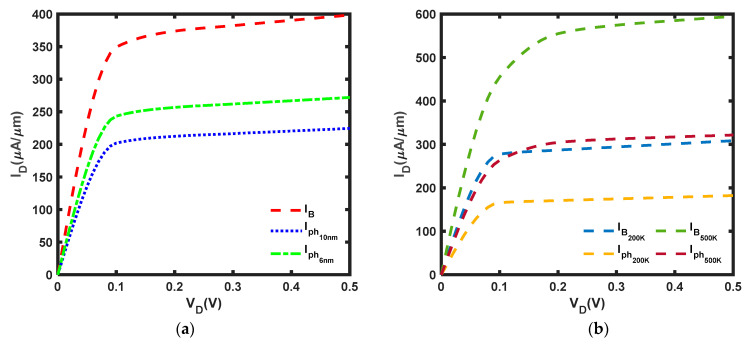
I–V characteristic of MoS_2_ FET at *V_G_* = 0.5 V with (**a**) *L* = 10 nm and *L* = 6 nm at T = 300 K (**b**) *L* = 10 nm at T = 200 K and T = 500 K: phonon scattering current, I_ph_, and ballistic current, I_B_.

The length scaling and applied voltage of this work were determined based on [37]. In Figure 6a, it is evident that the ballistic current has been significantly reduced by phonon scattering effects. It is observed that the current reduction is significant, with a 43.7% reduction at *L* = 10 nm and 31.8% at *L* = 6 nm. It was also noted that the current at *L* = 6 nm is 21.2% higher than that at *L* = 10 nm. As *L* approaches the phonon MFP, the percentage of current reduction decreases. Moreover, Figure 6b shows that the current decreases by 40.9% at T = 200 K and 45.9% at T = 500 K. Furthermore, the current at T = 500 K is 76.4% higher than the current at T = 200 K. As the temperature increases, the percentage of current drop has increased due to the phonon scattering governing at higher temperatures [49] and more electrons gaining adequate energy to undergo phonon scattering and scatter electrons even further [50]. In addition, the electrons experience more rigorous thermal vibration at a higher temperature, resulting in greater phonon scattering [51]. Furthermore, ref. [50] suggested that the scattering effects under elevated temperatures, which have a detrimental effect on device performance, are primarily dominated by optical phonons.

**Figure 7:**
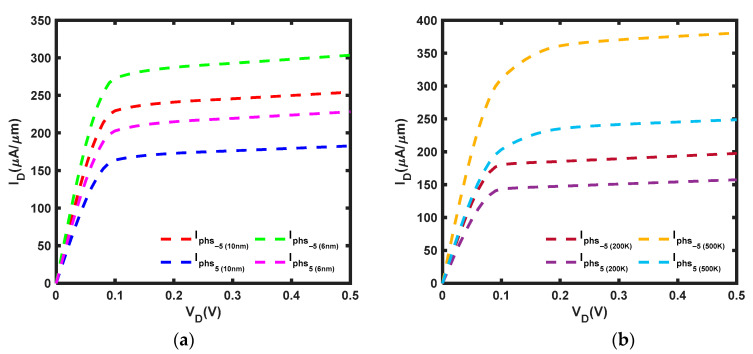
I–V characteristic of MoS_2_ FET under compressive strain, ε = −5%, tensile strain, ε = 5% and *V_G_* = 0.5 V with (**a**) *L* = 10 nm and *L* = 6 nm at T = 300 K (**b**) *L* = 10 nm at T = 200 K and T = 500 K: strain current, I_phs_.

As mentioned in [52], modifications in bond angle and bond length due to strain alter the effective masses of electrons, leading to energy separation in the *K* and *Q* valleys. Hence, this study adapts the effective mass at a different applied strain, ε, from [53]. A comparison of Figure 6a and Figure 7a reveals some notable distinctions. It is observed that a compressive strain of ε = −5% can offset the negative effects of phonon scattering, leading to a 13.3% and 11.6% amplification of the current at *L* = 10 nm and *L* = 6 nm, respectively. Moreover, the current under compressive strain at *L* = 6 nm is increased by 19.3% from that at *L* = 10 nm. Furthermore, Figure 7b revealed that the current is boosted by 8.34% at T = 200 K and 18.4% at T = 500 K, with the current under compressive strain at T = 500 K elevated by 92.7% compared to that at T = 200 K. In contrast, a tensile strain of ε = 5% cannot compensate for the impact of phonon scattering, leading to an 18.6% and 16.1% attenuation of the current at *L* = 10 nm and *L* = 6 nm, respectively. However, at *L* = 6 nm, the current under tensile strain is increased by 24.8% when compared to that at *L* = 10 nm. Furthermore, Figure 7b indicates that the current experienced a reduction of 13.5% under tensile strain at T = 200 K and a decrease of 22.6% at T = 500 K, with the current at T = 500 K enhanced by 57.9% relative to that at T = 200 K. According to [54], the reduction in the bandgap of MoS_2_ contributes to the enhancement of the current. Compressive strain resulted in a narrower bandgap compared to tensile strain. However, the current achieved under compressive strain is still significantly lower than the ballistic current.

**Figure 8:**
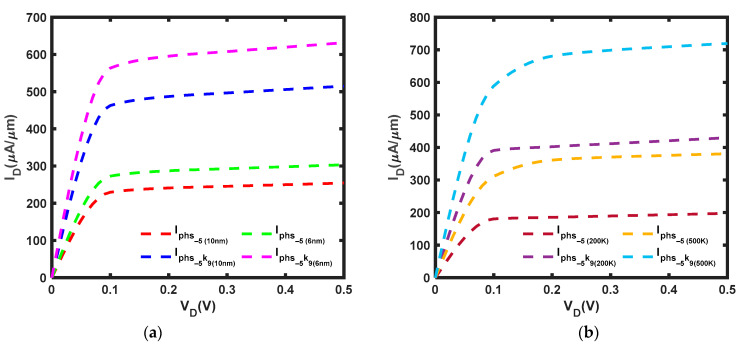
I–V characteristic of MoS_2_ FET at ε = −5%, k = 9 and *V_G_* = 0.5 V with (**a**) *L* = 10 nm and *L* = 6 nm at T = 300 K (**b**) *L* = 10 nm at T = 200 K and T = 500 K: high-k compressive strain current, I_phs-5k9_.

Al_2_O_3_ is a gate dielectric commonly used in MoS_2_ FET [55,56] because it has the ability to weaken scattering effects [57]. When compressive strain and k = 9 [58] are applied, the current increases significantly, even more than the ballistic current depicted in Figure 6a, with increments of 29.2% and 58.4% at *L* = 10 nm and *L* = 6 nm, respectively. In addition, Figure 8a presents an increase in current of 102.5% at *L* = 10 nm and 108.1% at *L* = 6 nm, with a 22.6% increase at *L* = 6 nm from *L* = 10 nm. Figure 8b shows that the current increases by 117.7% at T = 200 K and by 89.0% at T = 500 K, with an increase of 67.3% at T = 500 K from T = 200 K.

**Figure 9:**
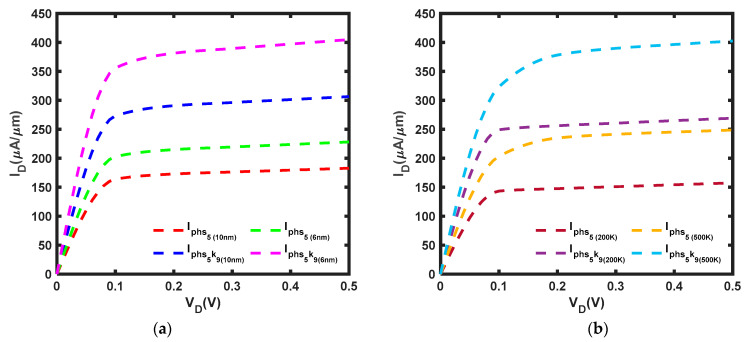
I–V characteristic of MoS_2_ FET at ε = 5%, k = 9 and *V_G_* = 0.5 V with (**a**) *L* = 10 nm and *L* = 6 nm at T = 300 K (**b**) *L* = 10 nm at T = 200 K and T = 500 K: high-k tensile strain current, I_phs5k9_.

According to Figure 9a, the current is improved by tensile strain but is still lower than the ballistic current. Specifically, the current is increased by 67.7% and 77.5% at *L* = 10 nm and at *L* = 6 nm, respectively, and there is also an increase of 32.1% from *L* = 10 nm to *L* = 6 nm. Next, Figure 9b depicted that the current is elevated by 70.9% at T = 200 K and 61.8% at T = 500 K. Moreover, the current is enhanced by 49.5% from T = 200 K to T = 500 K.

**Figure 10:**
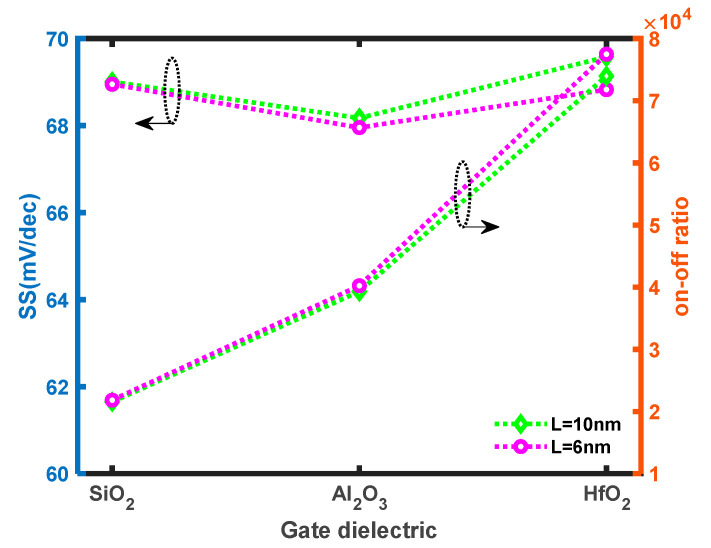
SS and on–off ratio of MoS_2_ FET at ε = −5% and *L* = 10 nm at T = 300 K.

Figure 10 illustrates that SS remains consistent across the different gate dielectrics and on–off ratios at different gate dielectrics: SiO_2_ (k = 3.9) [59], Al_2_O_3_ (k = 9) and HfO_2_ (k = 25) [60]. At *L* = 10 nm, the values of SS at SiO_2_, Al_2_O_3_ and HfO_2_ are 69.0 mV/dec, 68.2 mV/dec and 69.6 mV/dec, respectively, while the on–off ratio at SiO_2_, Al_2_O_3_ and HfO_2_ are 2.16 × 10^4^, 3.93 × 10^4^ and 7.40 × 10^4^. At *L* = 6 nm, the values of SS at SiO_2_, Al_2_O_3_ and HfO_2_ are 68.9 mV/dec, 68.0 mV/dec and 68.8 mV/dec, respectively, while the on–off ratio at SiO_2_, Al_2_O_3_ and HfO_2_ are 2.18 × 10^4^, 4.02 × 10^4^ and 7.75 × 10^4^, respectively. It is noteworthy that the SS remains relatively constant regardless of the type of dielectric employed. Conversely, the on–off ratio demonstrates an increasing trend with high-k dielectrics. This can be attributed to the shifting of the valence and conduction bands [61] and the enhancement of the fringing electric field within the device. These factors contribute to a higher on current and a lower off current, ultimately resulting in an improved on–off ratio [62]. Therefore, the utilization of high-k dielectrics is preferable in devices with shorter channel lengths because it increases the off current caused by the reduced distance between the source and drain, thereby enhancing the punch-through effect [63]. However, it is important to avoid high-k dielectric with excessively large values (k > 25) as they can lead to a stronger fringing electric field, thereby compromising gate control and failing to effectively suppress short channel effects [64]. In fact, the deposition of high-k dielectrics on MoS_2_ increases both the on and off currents due to the oxide vacancies in the materials. NH_3_ plasma treatment on high-k dielectrics can repair the vacancy defects and thus enhance the device performance [65,66].

**Figure 11:**
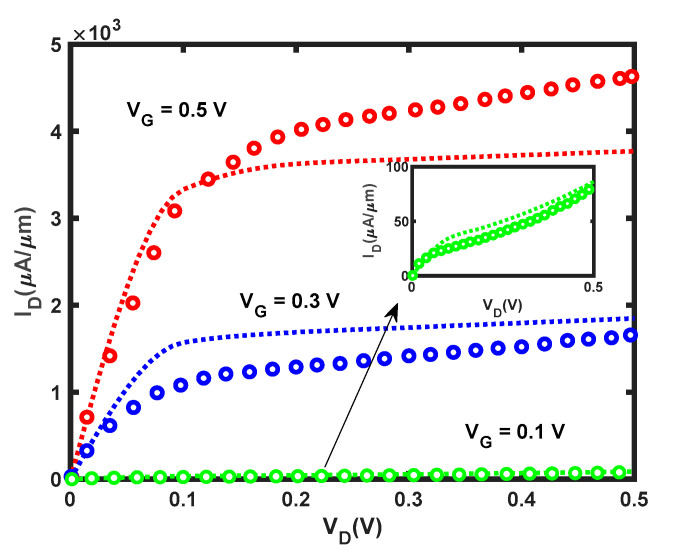
I–V characteristic of our study (dotted lines) and published study (open circles) [19] from *V_G_* = 0.1 V to *V_G_* = 0.5 V in the steps of 0.2 V.

The accuracy of our model is benchmarked against the published models and experimental data. Figure 11 depicts the comparison of our study and [19]; our model showed fairly good agreement with the published model despite the use of different approaches. Our model was assessed using experimental data [67,68], which showed similar values of SS and on–off ratio values. It is noted that SS of the device using SiO_2_ is considerably higher than the device utilizing high-k dielectrics. It is worth mentioning that decreasing the oxide thickness to a few nanometers can lead to a reduction in SS [69]. The physical dimensions and performance metrics of our model and published studies were tabulated in Table 1.

The performance of our model was also compared with that of a different 2D FET material from our previous studies, specifically a silicene nanoribbon field-effect transistor [37] which revealed comparable SS and on–off ratio values. While [37] showed SS and an on–off ratio of 67.9 mV/dec and 10^4^, our model exhibited SS and an on–off ratio of 68.3 mV/dec and 10^4^, respectively. These indicated that our model could serve as a guideline to realize the MoS_2_ FET. Furthermore, this comparison allowed us to evaluate the effectiveness and applicability of our model across various materials and device architectures, demonstrating its potential for broader application in the field of 2D FETs.

## 4. Conclusions

In conclusion, the phonon scattering and strain effects on MoS_2_ FET are investigated. The influence of phonon scattering on currents is reduced as the device’s channel length approaches the phonon MFP, but it increases at higher temperatures. Overall, phonon scattering reduces the ballistic current by more than 40% at different temperatures. At low energy levels (*E_FS_* < *U_scf_*), acoustic phonon scattering is more prominent, while optical phonon scattering becomes dominant as energy levels increase. At higher temperatures (500 K), the influence of optical phonon scattering exceeds that of acoustic phonon scattering. On the other hand, applying compressive strain increases the current by 13.3%, while tensile strain is reduced by 18.6% when compared to the phonon scattering current. The use of a high-k dielectric can enhance the current, even surpassing the ballistic current under compressive strain and in shorter channel length devices under tensile strain. The accuracy of the model was assessed and found to be in good agreement with published studies. Devices utilizing high-k dielectrics exhibit an improvement in SS. Nonetheless, caution should be exercised in choosing dielectrics with excessively high k values, as they may counteract the advantages offered by high-k materials. In summary, this study provides valuable insights into the influence of acoustic and optical phonons under various conditions, affecting the ballisticity of the device. Additionally, the introduction of strain has the potential to improve device performance. Moreover, high-k dielectrics can be employed to enhance the overall performance of the device. This study could be expanded by including other non-ballistic effects, such as interface trap charges, contact resistance and leakage currents. Furthermore, this modeling approach can be extended for utilization with other 2D materials.

## Figures and Tables

**Table 1:** Physical dimensions and performance metrics in our study and published studies.

	Dual-Gate Device		Single-Gate Device		Single-Gate Device	
Parameters	Our Study	Other Model [19]	Our Study	Experimental Data [67]	Our Study	Experimental Data [68]
Channel length, *L*	5 nm	5 nm	16 µm	16 µm	20 µm	20 µm
Temperature, T (K)	300	300	300	300	300	300
Dielectric constant, k	16	16	25	25	3.9	3.9
Oxide thickness, T_ox_ (nm)	2	2	10	10	90	90
SS (mV/dec)	153.5	157.0	76.4	77.6	359	360
On–off ratio	10^3^	10^3^	10^4^	10^4^	10^5^	10^5^

## Data Availability

The data that supports the findings of this study are available within the article and Appendix A.

## Appendix A

$$N_{S/D}=\frac{1}{A}\sum_{k_{x}>0,\;\;k_{y}}f\left(E-E_{FS/D}\right),$$

$$\;\;\;\;\;\;\;\;\;=\iint_{k_{x}>0,\;\;k_{y}}2\frac{d^{2}k}{{\left(2\pi \right)}^{2}}f\left(E-E_{FS/D}\right),$$

$$\;\;\;\;\;\;\;\;\;=\int_{-\infty }^{+\infty }dEf\left(E-E_{FS/D}\right)\frac{1}{2}\int_{S\left(E\right)}^{}\frac{dS}{2\pi^{2}}\frac{1}{\left|\nabla E\left(k\right)\right|}.$$

$S\left(E\right)$, dS, $dE/\left|\nabla E\left(k\right)\right|$ are the constant energy surface in *k*-space, the elemental area on this surface and the distance between the surface $S\left(E+dE\right)$ and $S\left(E\right)$. Defining the DOS as

$$D\left(E-U_{scf}\right)=\int_{S\left(E-U_{scf}\right)}^{}\frac{dS}{2\pi^{2}}\frac{1}{\left|\nabla E\left(k\right)\right|},$$

$$N_{S/D}=\frac{1}{2}\int_{-\infty }^{+\infty }D\left(E-U_{scf}\right)f\left(E-E_{FS/D}\right)dE.$$

Redefining the expression above,

$$N_{S/D}=\frac{1}{2}\int_{-\infty }^{+\infty }D\left(E\right)f\left(E-E_{FS/D}+U_{scf}\right)dE,$$

$$\;\;\;\;\;\;\;\;\;=\frac{1}{2}\int_{-\infty }^{+\infty }D\left(E\right)f_{S/D}\left(E\right)dE.$$

which is equivalent to Equation (5) or (6).
